# MapGL: inferring evolutionary gain and loss of short genomic sequence features by phylogenetic maximum parsimony

**DOI:** 10.1186/s12859-020-03742-9

**Published:** 2020-09-22

**Authors:** Adam G. Diehl, Alan P. Boyle

**Affiliations:** 1 Department of Computational Medicine and Bioinformatics, University of Michigan, Ann Arbor, MI USA; 2 Department of Human Genetics, University of Michigan, Ann Arbor, MI USA

**Keywords:** Phylogenetic analysis, Genomics, Genome evolution, Regulatory evolution, Genomic algorithms, Ancestral reconstruction

## Abstract

**Background:**

Comparative genomics studies are growing in number partly because of their unique ability to provide insight into shared and divergent biology between species. Of particular interest is the use of phylogenetic methods to infer the evolutionary history of cis-regulatory sequence features, which contribute strongly to phenotypic divergence and are frequently gained and lost in eutherian genomes. Understanding the mechanisms by which cis-regulatory element turnover generate emergent phenotypes is crucial to our understanding of adaptive evolution. Ancestral reconstruction methods can place species-specific cis-regulatory features in their evolutionary context, thus increasing our understanding of the process of regulatory sequence turnover. However, applying these methods to gain and loss of cis-regulatory features historically required complex workflows, preventing widespread adoption by the broad scientific community.

**Results:**

MapGL simplifies phylogenetic inference of the evolutionary history of short genomic sequence features by combining the necessary steps into a single piece of software with a simple set of inputs and outputs. We show that MapGL can reliably disambiguate the mechanisms underlying differential regulatory sequence content across a broad range of phylogenetic topologies and evolutionary distances. Thus, MapGL provides the necessary context to evaluate how genomic sequence gain and loss contribute to species-specific divergence.

**Conclusions:**

MapGL makes phylogenetic inference of species-specific sequence gain and loss easy for both expert and non-expert users, making it a powerful tool for gaining novel insights into genome evolution.

## Background

Comparative genomics uses sequence-level differences between species to gain insights into how genomes function and evolve [1]. According to Google Scholar, published comparative genomics studies have increased every year since 2009. These studies rely on the ability to detect and assign provenance to lineage-specific sequence variations at both the nucleotide level and at the level of larger-scale sequence insertions and deletions (indels). While species-specific indels are easily visible as gaps in pairwise alignments, gap presence alone tells us nothing about the underlying evolutionary processes by which it was created. Further analyses are necessary to differentiate between species-specific sequence insertions and loss of ancestral sequences. Understanding the mechanisms driving sequence divergence and their relationships to natural selection requires the ability to discern between these events. Ancestral reconstruction is a phylogenetic method by which observed states in outgroup species are used to infer the state in the most-recent common ancestor (MRCA) [2]. This inferred ancestral state can then be used to predict the evolutionary events leading to an indel. These methods can place indels in their proper evolutionary context, allowing much greater precision in hypothesis generation regarding the causes, underlying mechanisms, and downstream effects of sequence turnover.

However, ancestral reconstruction is a complex process and, while tools exist to reconstruct ancestral protein and DNA sequences [3–5] and ancestral genomes [6], no published tool exists to infer evolutionary gain and loss of short genomic sequence features. Doing so has historically relied on ad-hoc workflows involving multiple mapping steps to target and outgroup genomes and subsequent analysis with a combination of published genomics software and custom scripts (see [7] for example). While general phylogenetic inference software, such as TNT [8] and POY [9], may be used to perform the ancestral reconstruction steps of these pipelines, given a fixed phylogenetic tree, their proper application requires a level of specialist knowledge that may be prohibitive for the casual user. Furthermore, the complexity of these pipelines is exacerbated by the need to use multiple outgroup species in order to ensure the reliability of inferences [10], which comes at the cost of increased input and intermediate files, alignment and post-processing steps, disk and memory usage, and overall analysis time. These limitations represent a significant barrier to widespread application by the broad scientific community. MapGL addresses this problem by combining all mapping and phylogenetic inference steps into a single program with simple inputs and easily interpreted outputs.

## Implementation

**Fig. 1:**
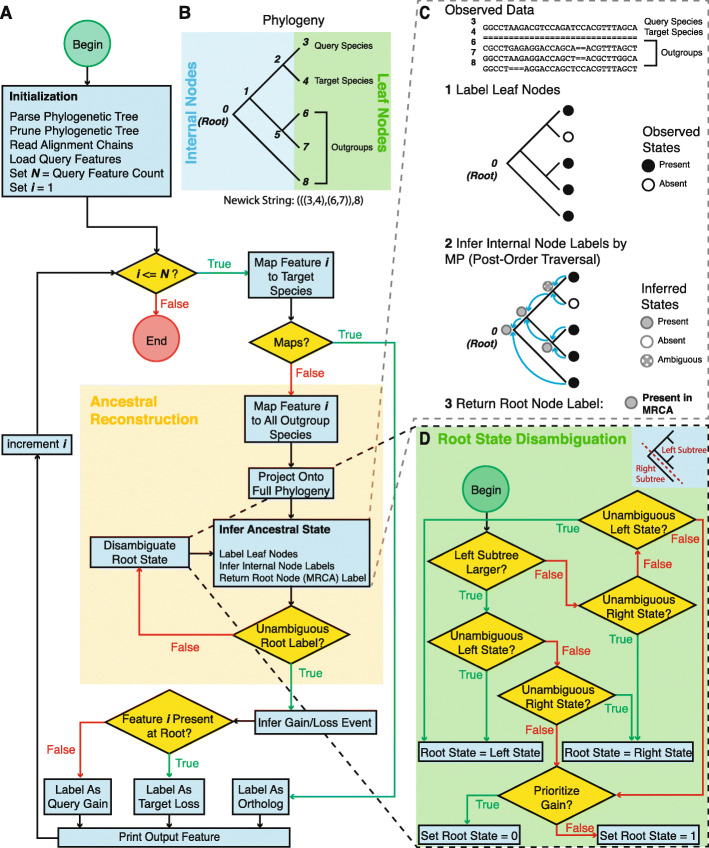
The mapGL algorithm. **a** Schematic outline for the mapGL algorithm. After initialization, the algorithm loops over query features, performing an initial mapping step against the target species. If the feature maps to the target species, it is labelled as an ortholog and written to output. If not, it enters the ancestral reconstruction stage. The feature is then mapped to each outgroup species in the full phylogeny and the corresponding leaves are labelled to indicate presence or absence. Internal labels are inferred based on the patterns observed at the leaf nodes (see Fig. 2). If the root state cannot be inferred unambiguously, root state disambiguation is performed as shown in panel D. Gain and loss events can then be inferred based on whether a feature is present at the root of the tree. The labelled feature is then written to output. This process is repeated until all query features are labelled. **b** Full phylogenetic tree describing evolutionary relationships between the query and target species (nodes 3 and 4) plus three outgroups (nodes 6–8). Query, target, and outgroup species occupy the leaf nodes of the tree. These are the only species for which we can directly observe sequence presence/absence. Internal nodes (0, 1, 2, and 5) represent ancestral species. **c** Since we cannot observe internal sequences directly, we must infer sequence presence/absence based on present-day observations from the leaf species. The core step of the ancestral reconstruction stage involves labelling all leaf nodes with their observed states and performing a post-order tree traversal to infer the states at internal nodes following the principle of maximum-parsimony (MP). The most-recent common ancestor (MRCA) occupies the root node (node 0), and the inferred state at this node is returned and used to predict whether query-specific sequences were gained in the query genome or lost from the target genome (see Fig. 2a-b) for example). **(D)** In cases when the root state cannot be resolved, the root state is disambiguated following a simple decision tree. In the first step, the larger of the left and right subtrees is chosen. If the state at the base of this tree is unambiguous, the root state is set to the corresponding state. Otherwise, we check the state at the base of the opposite subtree. If this node is not a leaf node and is labeled with an unambiguous state, we set the root state to the corresponding state. If neither left nor right subtrees have an unambiguous root state, or if the only unambiguous descendant node is a leaf node, the root state is chosen based on the –priority parameter. If this is set to “gain,” the root state defaults to 0 (sequence absence). If it is set to “loss,” the root state defaults to 1 (sequence presence)

**Fig. 2:**
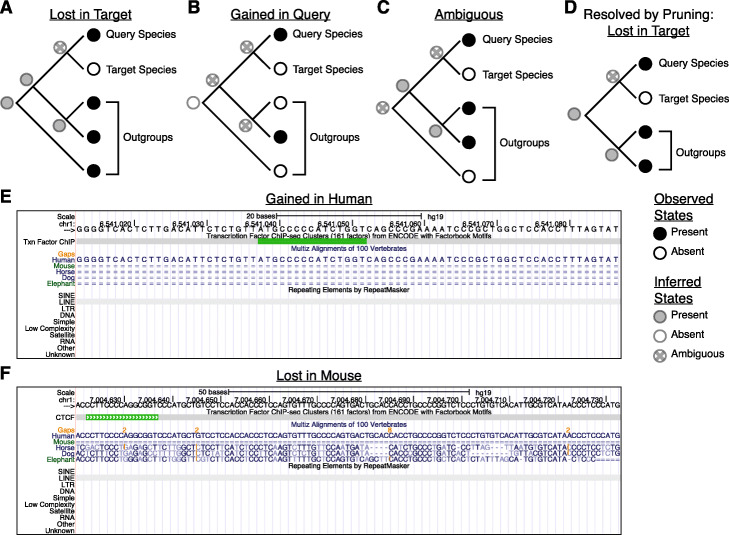
Example gain and loss inferences based on ancestral state reconstruction. **a** Example sequence loss in the target species based on presence in the most-recent common ancestor (MRCA), at the root of the tree. **b** Example sequence gain in the query species. Sequence absence is the most parsimonious inferred ancestral state as it invokes two independent sequence gains rather than three independent losses necessary to explain the observed data. **d** The phylogeny in C may be resolved by choosing an unambiguous state from one of the two nodes directly above the root. In the case of the tree shown, this is equivalent to pruning the outermost outgroup and rerooting the tree. The inferred ancestral state, then, is presence of an orthologous sequence and sequence loss in the target species is called. **e** UCSC Genome Browser track for a region in the human genome (hg19 build) labelled by MapGL as a human-specific gain. The region contains a CTCF binding annotation residing in a primate-specific insertion of an L2a LINE transposable element. **f** UCSC Genome Browser track for a region in the human genome labelled by MapGL as a mouse-specific loss. This region also contains a CTCF binding site residing within an L2a LINE element, but one that was inserted prior to divergence of Atlantogenata and Boreoeutheria species

MapGL applies a simple phylogenetic inference approach based on Wagner parsimony [11] to infer the evolutionary history of short genomic sequence features from a query genome relative to a target genome (Fig. 1a). This approach seeks to minimize the number of state-changes (i.e., sequence gains and losses) necessary to explain the pattern of sequence presence/absence in a multiple-alignment of contemporary sequences. Briefly, for each query feature, an initial mapping step to the target genome determines whether an orthologous sequence exists. If so, the sequence is labeled as an ortholog and written to output. Otherwise, the ancestral state is inferred by projecting observed data from the query, target, and outgroup species onto a precomputed, fixed phylogeny describing the evolutionary relationships between all present-day and ancestral species (Fig. 1b). Ancestral state inference proceeds using an adaptation of Fitch’s Algorithm [12], whereby the most-likely ancestral state is chosen to minimize the total number of gain/loss events required to explain the pattern of sequence presence/absence in the observed data [12] (Fig. 1c). Query sequences are first mapped to each outgroup and state labels recorded at the corresponding leaf nodes: “1” if an orthologous sequence exists or “0” if not. Next, a post-order traversal is performed to infer states at all internal nodes, choosing the most-frequent state observed among child nodes, or storing the union set of observed symbols in case of ties. The inferred state at the root node, representing the MRCA, is then used to infer whether sequences were gained on the branch leading to the query species (i.e., were absent in the MRCA) or lost on the branch leading to the target species (i.e., were present in the MRCA) (Fig. 2a-b).

In cases where the root state cannot be resolved, we set the root state equal to the unambiguous descendant state supported by the subtree with the most leaves. In the case of a single terminal outgroup species, where the right subtree has only one leaf, this is equivalent to pruning the most-terminal outgroup at the branch directly above the root (Fig. 1d, Fig. 2c-d). When this fails, we choose the root state based on the value of the –priority (−p) parameter that prioritizes sequence gain or loss. Setting this to “gain” is equivalent to assigning a lower evolutionary cost to sequence insertions compared to deletions. The root state will then default to “0” in ambiguous cases and sequence presence in descendant nodes will be interpreted as sequence gain. Setting this to “loss” will have the opposite effect. This behavior can be disabled via the –no_prune (−n) option, in which case such sequences will be labeled as “ambiguous.” Optionally, an additional pre-order tree traversal can be performed to infer and report all branch(es) on which sequence gain/loss events occurred throughout the phylogeny.

MapGL takes as inputs a set of genomic query features in BED format, a set of liftover chains [13] corresponding to the target and outgroup species, and a Newick tree describing the full phylogeny. The program is self-documenting and ships with a small example dataset. MapGL can be tuned through several command-line options. The most important of these are –threshold (−t) and –gap (−g). The -t < FLOAT> option specifies the amount of overlap required between query features and target chains, expressed as the fraction of query nucleotides overlapping a target chain, to call a match as a function of the query element length. The default value of 0.0 is equivalent to requiring a single-base overlap while a value of 1 would enforce only full-length alignments. The -g < INT> option specifies the tolerance for gaps and takes an integer specifying the maximum tolerable gap length, in nucleotides, as its argument. The default value of −1 indicates that gaps of any length are allowed. The default values maximize the algorithm’s ability to find orthologous sequences, making gain and loss predictions very conservative. However, these values may lead to erroneous ortholog predictions in cases where aligned features contain embedded indels, may be overly conservative when comparing closely related genomes, and may lead to underprediction of sequence gains/losses in poorly aligned regions. Specifying a non-zero mapping threshold and a gap tolerance near the average gap length in such regions may increase the sensitivity of MapGL to detect gain and loss events, albeit at the expense of reducing its sensitivity for orthologs. Nonetheless, as the prototypical use of this program is to identify whole sequence features that have undergone species-specific gain or loss, the default values are likely reasonable for most users.

MapGL is free and open-sourced under the MIT License. It is based on bnMapper [14], which maps genomic sequences across species based on liftover chains [13]. We retained the core mapping steps from bnMapper with one modification: while bnMapper drops alignments that are split across chains, we keep the longest alignment among all chains represented. This behavior can be controlled with the –drop_split (−d) option. We extended this framework by adding the ability to map against multiple liftover chains to incorporate target and outgroup species and added the ancestral reconstruction steps outlined above. Tree parsing and phylogenetic methods were adapted from the python-newick package (https://pypi.org/project/newick/). MapGL is compatible with MacOS and most Unix-like operating systems, and is available for installation through PyPI and Bioconda, with source code available from the GitHub repository (https://github.com/adadiehl/mapGL).

We present three case studies spanning a wide variety of phylogenetic tree topologies and evolutionary distances to establish the performance of MapGL. We obtained CTCF binding locations in one of two query species: human and *D. melanogaster*. Human data were compared to mouse, a distant target species, in the mammalian analysis (Fig. S1A), and chimpanzee, a closely related target species, in the primate analysis (Fig. S1C). *D. melanogaster* data were compared to *D. simulans*, which are roughly three times more divergent than human and chimpanzee, in the invertebrate analysis (Fig. S1E). Human CTCF data were originally analyzed as part of a study on the effects of transposable element (TE) activity on CTCF binding site content in mammalian genomes [15]. In keeping with this, dog, horse, and elephant were chosen as outgroups to yield phylogenetic tree that encompasses two major groups of mammals: Atlantogenata and Boreoeutheria, spanning the primary activity periods for several TE types associated with CTCF binding (Fig. S1A). Methods for all three analyses are described in detail within the Supplementary Methods.

## Results

Results of the mammalian analysis show that roughly 1/3 of human CTCF binding sites are not found in mouse, either as a result of human-specific gain or mouse-specific loss (Fig. S1B). We show two representative examples in Fig. 2e-f, noting that that both show the expected patterns of sequence presence/absence for their respective labels. Importantly, these two predictions demonstrate how even sites with similar functional annotations can have very different evolutionary origins. Although both these regions contain human-specific CTCF binding sites derived from L2a retrotransposon insertions, these insertions occurred at very distant points in evolutionary history. The feature shown in in Fig. 2e originated from an L2a insertion that occurred after primate-rodent divergence while the region in Fig. 2f originated from an L2a insertion that occurred very early in placental mammal evolution, prior to the divergence of Atlantogenata and Boreoeutheria, and was subsequently lost from the mouse genome. Thus, MapGL can discern between elements with different evolutionary histories, leading to distinct interpretations regarding their roles in regulatory innovation.

Consistent with the relatively small evolutionary distance between human and chimpanzee, we observed a substantially lower rate of turnover in CTCF binding sequence content in the primate analysis relative to the mammalian analysis (Fig. S1C). However, the expected patterns of sequence presence/absence were still evident in multiple sequence alignments for gain and loss predictions (Fig. S2A-B). Likewise, the invertebrate analysis yielded similar results. Interestingly, the level of CTCF binding turnover observed in *D. melanogaster* compared to *D. simulans* (Fig. S1F) was very close to the rate observed in comparison between human and chimpanzee (Fig. S1D) despite being roughly threefold more divergent at the sequence level. This may result from strong selection to maintain a compact genome, leading to a low tolerance for genomic insertions [16]. Nonetheless, representative gain and loss predictions from *D. melanogaster* exhibit the expected characteristics in multiple sequence alignments (Figs. S2C-D).

## Conclusions

Comparative genomics relies on the ability to infer the creative mechanisms and downstream effects of lineage-specific sequence divergence. However, even sequences with similar properties can have very different evolutionary histories. This ambiguity compromises our ability to determine how lineage-specific gain and loss of short sequence features relates to conserved and divergent functions. Placing species-specific sequence features in their proper evolutionary context enables a deeper understanding of functional divergence but requires application of phylogenetic methods that have not been previously integrated into a single piece of software. MapGL answers this need, opening these methods to a much broader user base.

While query and target species will have generally been chosen prior to the decision to apply MapGL, the choice of appropriate outgroups should also be considered carefully, as it is critical in optimizing the performance of MapGL. Thus, we offer some general guidelines for outgroup selection. First, we recommend using a minimum of two outgroups, while three or more is better. Because MapGL utilizes a known, fixed phylogeny to describe species relationships, errors in the tree topology may lead to erroneous gain/loss inferences. Therefore, we advise choosing species for which a reliable phylogeny is available. In order to ensure sufficient data to resolve between competing gain/loss scenarios, the amount of evolutionary distance captured within the outgroup subtree (i.e., the sum of the branch lengths connecting all leaves and internal nodes) should equal or exceed the distance between the query/target clade. Likewise, to avoid biasing gain/loss predictions toward either the query or target branch, the overall distance between the nearest outgroup and the root of the query/target clade should be at least as long as the shorter of the query or target branch. Finally, the mechanisms involved in creating indel sequences of interest should be considered as they may dictate the evolutionary distances necessary to accurately resolve competing gain/loss scenarios. For example, sequence turnover that occurs gradually, such as accumulation of small indels over many generations, may require relatively distant outgroups for reliable inference. By contrast, processes that occur within a relatively short evolutionary timespan, such as individual transposon dispersal events, may be resolvable using more closely related outgroups. It is also important to note that MapGL depends on the accuracy of chain files used to determine sequence presence/absence in target and outgroup species. Intuitively, alignment errors can create erroneous gaps in alignment chains, leading to erroneous gain/loss predictions. Since these errors are more prevalent in alignments between low-coverage/low-quality genome assemblies, these metrics should be considered in selecting outgroup species.

Similarly, inferences within difficult-to-align regions, including simple sequence repeats and repetitive elements, may be of lesser quality than those elsewhere. In some cases, difficult-to-align sequences may manifest themselves as regions with many apparent substitutions and indels. As discussed in the Implementation section, predictive accuracy within such regions may be improved through careful tuning of the –gap (−g) and –threshold (−t) options. We note several use cases where it may be beneficial to use non-default values for the -g and -t parameters, but this list is far from exhaustive: 1) comparisons between closely-related query and target species (e.g., human and chimp); 2) labelling query sequences that fall within difficult-to-align regions with high rates of alignment error; 3) classifying sequences created by indel processes prone to creating multiple, closely-spaced indels; 4) maximizing the specificity of ortholog predictions at the expense of decreasing predictive accuracy for gain/loss events.

MapGL was designed to resolve the evolutionary histories of indels between a pair of query and target species. Therefore, by design, its resolution in placing gain and loss events in evolutionary time is inherently limited. While the program does not prohibit intermediate species in the phylogeny, beyond providing additional data from which to predict the ancestral state, their inclusion will not improve the temporal resolution or quality of gain/loss predictions. However, we show that the current implementation of MapGL performs well in the specific tasks for which it was designed and should be sufficient for most anticipated use cases. We show that MapGL performs well over a wide range of evolutionary distances and tree topologies in both mammalian and invertebrate datasets, establishing MapGL as a simple and robust method to infer the evolutionary histories of short sequence features.

### Availability and requirements

Project name: MapGL

Project home page: https://github.com/adadiehl/mapGL. (Python package available through PyPi, and Bioconda)

Operating systems: MacOS, Linux

Programming language: Python

Other Requirements: None

License: MIT

Restrictions: None

## Supplementary information

**Additional file 1: Table S1.** Datasets. **Figure S1.** Phylogenetic trees and gain/loss statistics for CTCF binding sites in mammalian, primate, and invertebrate phylogenies. **Figure S2.** Representative gain and loss predictions from the primate and invertebrate analyses.

## Data Availability

All primary datasets used in this analysis, listed in Table S1, are freely and publicly available from their listed sources. All derived datasets and results generated in the current study can be reproduced using scripts available through the GitHub repository for this analysis: https://github.com/adadiehl/MapGL_analysis.

## Abbreviations

Indel: Insertion or deletion of genomic sequence
MRCA: Most-Recent Common Ancestor
TE: Transposable Element
