# Supplementary material for: MapGL: inferring evolutionary gain and loss of short genomic sequence features by phylogenetic maximum parsimony
Source: BMC Bioinformatics. 2020 Sep 22;21:416. doi: 10.1186/s12859-020-03742-9 (PMC7510305; doi:10.1186/s12859-020-03742-9)
Supplement: Supplementary file 1 — Additional file 1: Table S1. Datasets. Figure S1. Phylogenetic trees and gain/loss statistics for CTCF binding sites in mammalian, primate, and invertebrate phylogenies. Figure S2. Representative gain and loss predictions from the primate and invertebrate analyses. [file 12859_2020_3742_MOESM1_ESM.pdf]

Supplementary Material for:

MapGL: Inferring evolutionary gain and loss of short genomic sequence features by phylogenetic maximum parsimony.

Adam G Diehl[1†*] and Alan P Boyle[1,2]
1. Department of Computational Medicine and Bioinformatics, University of Michigan, Ann Arbor, MI
2. Department of Human Genetics, University of Michigan, Ann Arbor, MI
† To whom correspondence should be addressed.
*e-mail: adadiehl@umich.edu

Table of Contents:

Table S1: Datasets
Supplementary Methods
Supplementary References
Figure S1: Phylogenetic trees and gain/loss statistics for CTCF binding sites in mammalian, primate, and invertebrate phylogenies
Figure S2: Representative gain and loss predictions from the primate and invertebrate analyses

Table S1: Datasets

Accession	Type	Target	Genome	Cell	URL	Description
ENCFF085HTY	ChIP-seq	CTCF	hg19	K562	https://www.encodeproject.org	CTCF ChIP-seq
ENCFF002CEL	ChIP-seq	CTCF	hg19	K562	https://www.encodeproject.org	CTCF ChIP-seq
ENCFF738TKN	ChIP-seq	CTCF	hg19	K562	https://www.encodeproject.org	CTCF ChIP-seq
ENCFF002DDJ	ChIP-seq	CTCF	hg19	K562	https://www.encodeproject.org	CTCF ChIP-seq
ENCFF002DBD	ChIP-seq	CTCF	hg19	K562	https://www.encodeproject.org	CTCF ChIP-seq
ENCFF710VEH	ChIP-seq	CTCF	hg19	GM12878	https://www.encodeproject.org	CTCF ChIP-seq
ENCFF096AKZ	ChIP-seq	CTCF	hg19	GM12878	https://www.encodeproject.org	CTCF ChIP-seq
ENCFF002DAJ	ChIP-seq	CTCF	hg19	GM12878	https://www.encodeproject.org	CTCF ChIP-seq
ENCFF963PJY	ChIP-seq	CTCF	hg19	GM12878	https://www.encodeproject.org	CTCF ChIP-seq
ENCFF123VEZ	ChIP-seq	CTCF	dm3	S2	https://www.encodeproject.org	CTCF ChIP-seq
	Alignment Chain	mm9	hg19		http://hgdownload.soe.ucsc.edu/goldenPath/hg19/liftOver/hg19ToMm9.over.chain.gz	Human to Mouse alignment chain
	Alignment Chain	canFam2	hg19		http://hgdownload.soe.ucsc.edu/goldenPath/hg19/liftOver/hg19TocanFam2.over.chain.gz	Human to Dog alignment chain
	Alignment Chain	equCab2	hg19		http://hgdownload.soe.ucsc.edu/goldenPath/hg19/liftOver/hg19ToEquCab2.over.chain.gz	Human to Horse alignment chain
	Alignment Chain	loxAfr3	hg19		http://hgdownload.soe.ucsc.edu/goldenPath/hg19/liftOver/hg19ToLoxAfr3.over.chain.gz	Human to Elephant alignment chain
	Alignment Chain	panTro4	hg19		http://hgdownload.soe.ucsc.edu/goldenPath/hg19/liftOver/hg19ToPanTro4.over.chain.gz	Human to Chimpanzee alignment chain
	Alignment Chain	rheMac3	hg19		http://hgdownload.soe.ucsc.edu/goldenPath/hg19/liftOver/hg19ToRheMac3.over.chain.gz	Human to Rhesus alignment chain
	Alignment Chain	macFas5	hg19		http://hgdownload.soe.ucsc.edu/goldenPath/hg19/liftOver/hg19ToMacFas5.over.chain.gz	Human to Crab-eating Macaque alignment chain
	Alignment Chain	calJac3	hg19		http://hgdownload.soe.ucsc.edu/goldenPath/hg19/liftOver/hg19ToCalJac3.over.chain.gz	Human to Marmoset alignment chain
	Alignment Chain	saiBol1	hg19		http://hgdownload.soe.ucsc.edu/goldenPath/hg19/liftOver/hg19ToSaiBol1.over.chain.gz	Human to Squirrel Monkey alignment chain
	Alignment Chain	droSim1	dm3		http://hgdownload.soe.ucsc.edu/goldenPath/dm3/liftOver/dm3ToDroSim1.over.chain.gz	D. melanogaster to D. simulans alignment chain
	Alignment Chain	droAna3	dm3		http://hgdownload.soe.ucsc.edu/goldenPath/dm3/liftOver/dm3ToDroAna1.over.chain.gz	D. melanogaster to D. ananassae alignment chain
	Alignment Chain	dp3	dm3		http://hgdownload.soe.ucsc.edu/goldenPath/dm3/liftOver/dm3ToDp3.over.chain.gz	D. melanogaster to D. pseudoobscura alignment chain
	Alignment Chain	anoGam1	dm3		http://hgdownload.soe.ucsc.edu/goldenPath/dm3/liftOver/dm3ToAnoGam1.over.chain.gz	D. melanogaster to A. gambiae alignment chain
	Phylogenetic Tree				http://hgdownload.soe.ucsc.edu/goldenPath/hg19/multiz100way/hg19.100way.nh	Phylogenetic tree of 100 mammalian species
	Phylogenetic Tree				http://hgdownload.soe.ucsc.edu/goldenPath/dm6/multiz27way/dm6.27way.nh	Phylogenetic tree of 27 insect species

Supplementary Methods

For our pilot analysis, we applied MapGL to a mammalian phylogeny with human (hg19) as the query species and mouse (mm9) as the target species. Dog (canFam2), horse (equCab2), and elephant (loxAfr3) were chosen as outgroups (Fig. S1A). This phylogeny includes species from two major groups of placental mammals: Atlantogenata and Boreoeutheria, and was selected to span an evolutionary distance encompassing the estimated activity dates of major transposable element families under investigation as sources of CTCF binding sites in the human and mouse genomes. Other considerations in selecting outgroup species were genome assembly and alignment quality, branch lengths separating individual outgroup species, and overall branch length separating outgroups from the human-mouse subtree. Horse and dog were chosen because their genome assemblies are of high quality, and they have a similar level of divergence relative to human and mouse. Elephant was chosen as a distal outgroup based on the high quality of its genome assembly and distance from both the human-mouse and horse-dog subtrees, leading to an overall outgroup tree length nearly equal to the human-mouse subtree. For comparison, we applied MapGL to two alternative phylogenies: a primate phylogeny spanning a much shorter evolutionary distance than the mammalian phylogeny, and an invertebrate phylogeny spanning a much larger evolutionary distance than either of the two other phylogenies.

Alignment chains and phylogenetic trees were obtained from the UCSC Genome Browser (1) download portal (Table S1). The newick tree including only human, mouse, dog, horse, and elephant, was extracted from the 100-way vertebrate phylogeny using tree_doctor, and trees were visualized with draw_tree, both from the Phast software package (2). As part of the parent analysis (3), CTCF ChIP-seq data for two human immune cell lines were obtained from the ENCODE project portal (4) (Supplementary Table 1). These were merged into a union dataset with bedtools merge (5). MapGL was then applied to the union CTCF dataset to label each binding site as an ortholog, gain, or loss. Statistics were gathered in and visualized in R. Results are summarized in Fig. S1B and representative gain and loss events are presented in Figure 2E-F of the main text.

The primate phylogeny consisted of human (hg19) and chimpanzee (panTro4) as the query and target species with rhesus macaque (rheMac3), crab-eating macaque (macFas5), marmoset (calJac3), and squirrel monkey (SaiBol1) as outgroups (Fig. S1C). For this test, we utilized the same human CTCF ChIP-seq dataset used in the mammalian analysis. The primate phylogenetic tree was extracted from the 100-way vertebrate phylogeny as described above, and sites were analyzed with MapGL using default options. Results are summarized in Fig. S1D and representative gain and loss events are depicted in Fig. S2A-B.

The invertebrate phylogeny consisted of four Drosophila species: D. melanogaster, D. simulans, D. ananassae, and D. pseudoobscura, and mosquito (A. gambiae) (Fig. S1E). We used CTCF ChIP-seq data for D. melanogaster, obtained from the ENCODE project, as a query dataset, and D. simulans as the target, with the remaining three species as outgroups. As no phylogenetic tree was available from UCSC for the dm3 assembly, we retrieved the 27-way insect phylogeny for the dm6 assembly from the UCSC Genome Browser download portal (1). We extracted the five species used in the invertebrate comparison using tree_doctor (2) and relabeled the “dm6” and “droPse3” nodes as “dm3” and “dp3”, respectively, to match assembly names used in this analysis. The resulting tree was visualized using draw_tree (2). CTCF binding sites were then analyzed with MapGL using default options. Data were analyzed for visualization in R, with results presented in Fig. S1F. Typical gain and loss events are presented in Fig. S2C-D.

Supplementary References

1. Kent WJ, Sugnet CW, Furey TS, Roskin KM, Pringle TH, Zahler AM, et al. The human genome browser at UCSC. Genome Res. 2002 Jun;12(6):996–1006.

2. Hubisz MJ, Pollard KS, Siepel A. PHAST and RPHAST: phylogenetic analysis with space/time models. Brief Bioinformatics [Internet]. 2011 Jan;12(1):41–51. Available from: http://www.ncbi.nlm.nih.gov/pubmed/21278375

3. Diehl AG, Ouyang N, Boyle AP. Transposable elements contribute to cell and species-specific chromatin looping and gene regulation in mammalian genomes. Nature Communications. Nature Publishing Group; 2020 Apr 14;11(1):1796–18.

4. Consortium EP. The ENCODE (ENCyclopedia of DNA elements) project. Science. 2004 Oct;306(5696):636–40.

5. Quinlan AR, Hall IM. BEDTools: a flexible suite of utilities for comparing genomic features. Bioinformatics. 2010 Mar;26(6):841–2.

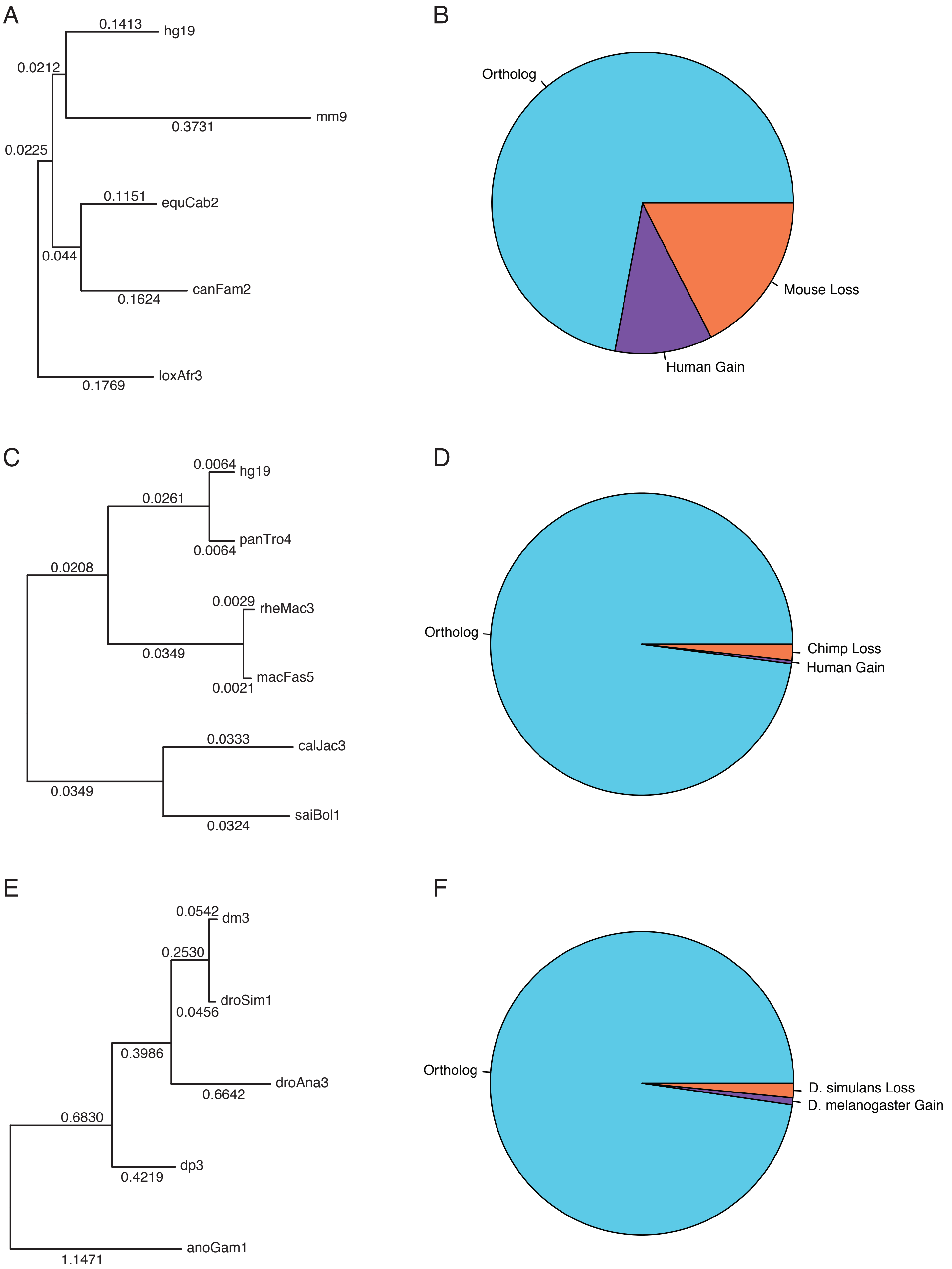

A
0.1413
hg19
0.0212
0.3731
mm9
0.0225
0.1151
equCab2
0.044
0.1624
canFam2
loxAfr3
0.1769
B
Ortholog
Mouse Loss
Human Gain
C
0.0064
hg19
0.0261
panTro4
0.0064
0.0208
0.0029
rheMac3
0.0349
macFas5
0.0021
0.0333
calJac3
0.0349
saiBol1
0.0324
D
Ortholog
Chimp Loss
Human Gain
E
0.0542
dm3
0.2530
droSim1
0.0456
0.3986
droAna3
0.6642
0.6830
dp3
0.4219
anoGam1
1.1471
F
Ortholog
D. simulans Loss
D. melanogaster Gain

Figure S1: Phylogenetic trees and gain/loss statistics for CTCF binding sites in mammalian, primate, and invertebrate phylogenies. For all trees, branch lengths are given in units of substitutions per site and leaf nodes are labelled with the genome assembly name corresponding to their species. **A)** Phylogenetic tree used for the mammalian analysis. Human=hg19, mouse=mm9, dog=canFam2, horse=equCab2, elephant=loxAfr3. **B)** Proportion of human CTCF binding site annotations labelled as orthologs, gains, and losses in the mammalian analysis. **C)** Phylogenetic tree used in the primate analysis. Human=hg19, chimpanzee=panTro4, rhesus macaque=rheMac3, crab-eating macaque=macFas5, marmoset=calJac3, squirrel monkey=saiBol1. **D)** Proportion of human CTCF binding site annotations labelled as orthologs, gains, and losses in the primate analysis. **E)** Phylogenetic tree used in the invertebrate analysis. D. melanogaster=dm3, D. simulans=droSim1, D. ananassae=droAna3, D. pseudoobscura=dp3, A. gambiae=anoGam1. **F)** Proportion of D. melanogaster CTCF binding site annotations labelled as orthologs, gains, and losses in the invertebrate analysis.

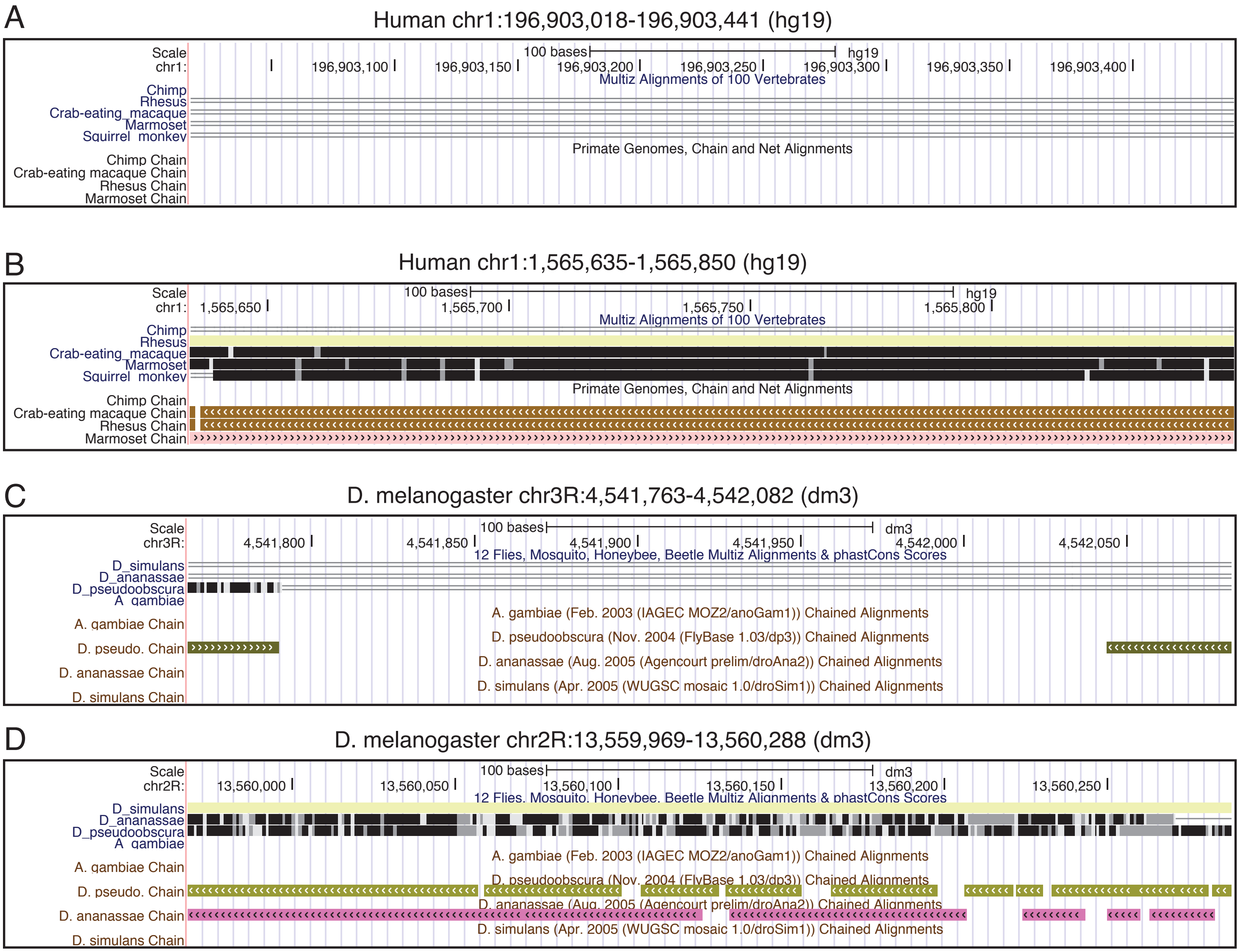

Figure S2: Representative gain and loss predictions from the primate and invertebrate analyses. Images were captured from the UCSC Genome Browser {Kent:2002ch}. Primate predictions are referenced to the hg19 genome build while invertebrate predictions are referenced to the dm3 genome build. **A)** A typical human-specific sequence gain prediction found on human chromosome (chr1:196,903,018-196,903,441). Note absence of sequence annotations within chimp, rhesus, crab-eating macaque, rhesus macaque, marmoset, and squirrel monkey multiple alignment and chain tracks. **B)** A typical chimpanzee-specific sequence loss prediction found on human chromosome 1 (chr1:1,565,635-1,565,850). Note the absence of sequence in chimp sequence in the multiple alignment and chain tracks, whereas sequence alignments and chains are present for all outgroup species. **C)** Representative D. melanogaster sequence gain prediction found on chromosome 3R (chr3R:4,541,763-4,542,082). Aside from small flanking fragments in D. pseudoobscura, all outgroup species lack sequence in this region, making D. melanogaster-specific sequence gain the most likely explanation for the observed data. **D)** Representative D. simulans sequence loss prediction found on D. melanogaster chromosome 2R (chr2R:13,559,969-13,560,288). Sequence is present in D. melanogaster, D. ananassae, and D. pseudoobscura, but absent in D. simulans and A. gambiae, making sequence loss on the branch leading to D. simulans the most parsimonious explanation for the observed data.
